# Multiplexed neural recording along a single optical fiber via optical reflectometry

**DOI:** 10.1117/1.JBO.21.5.057003

**Published:** 2016-05-19

**Authors:** Samuel G. Rodriques, Adam H. Marblestone, Jorg Scholvin, Joel Dapello, Deblina Sarkar, Max Mankin, Ruixuan Gao, Lowell Wood, Edward S. Boyden

**Affiliations:** a MIT Media Lab, E15-421, 20 Ames Street, Cambridge, Massachusetts 02139, United States; b Massachusetts Institute of Technology, Department of Physics, 4-315, 77 Massachusetts Avenue, Cambridge, Massachusetts 02139, United States; c Harvard University, Department of Chemistry and Chemical Biology, 12 Oxford Street Cambridge, Massachusetts 02138, United States; d Bellevue, Washington, United States; e MIT Media Lab and McGovern Institute, Departments of Brain and Cognitive Science and Biological Engineering, United States

**Keywords:** reflectometry, neural recording, fiber-optic, electro-optic, nanophotonics

## Abstract

We introduce the design and theoretical analysis of a fiber-optic architecture for neural recording without contrast agents, which transduces neural electrical signals into a multiplexed optical readout. Our sensor design is inspired by electro-optic modulators, which modulate the refractive index of a waveguide by applying a voltage across an electro-optic core material. We estimate that this design would allow recording of the activities of individual neurons located at points along a 10-cm length of optical fiber with 40-μm axial resolution and sensitivity down to 100  μV using commercially available optical reflectometers as readout devices. Neural recording sites detect a potential difference against a reference and apply this potential to a capacitor. The waveguide serves as one of the plates of the capacitor, so charge accumulation across the capacitor results in an optical effect. A key concept of the design is that the sensitivity can be improved by increasing the capacitance. To maximize the capacitance, we utilize a microscopic layer of material with high relative permittivity. If suitable materials can be found—possessing high capacitance per unit area as well as favorable properties with respect to toxicity, optical attenuation, ohmic junctions, and surface capacitance—then such sensing fibers could, in principle, be scaled down to few-micron cross-sections for minimally invasive neural interfacing. We study these material requirements and propose potential material choices. Custom-designed multimaterial optical fibers, probed using a reflectometric readout, may, therefore, provide a powerful platform for neural sensing.

## 1 Introduction

The extracellular electrode is a classic neural recording technology. The electrode is essentially a conductive wire, insulated except at its tip, placed in the extracellular medium as close as possible to a neuron of interest, where it samples the local voltage relative to a common reference in the brain.^1^^,^^2^ This extracellular voltage differential is typically on the order of 100  μV in response to an action potential from a nearby neuron^3^ and decays over a distance on the order of 100  μm. Note that the “transmembrane” voltage during an action potential is much larger, on the order of 100 mV.

The virtues of the electrode are twofold. First, the technique can reach single neuron precision by virtue of the electrode being inserted close to the measured neuron. Second, compared to optical methods, no exogenous contrast agents (i.e., genetically encoded fluorescent proteins, voltage sensitive nanoparticles, chemical dyes) are necessary: the endogenously generated electric currents in the brain are sensed directly in the form of a voltage. Ideally, for a neurotechnology to be medically valuable for a large number of human patients, it should not require modification of the neuron.

Yet, while multielectrode arrays allow the insertion of many electrodes into a brain, electrodes have limitations^3^ in scaling to the simultaneous observation of large numbers of neurons. The bandwidth of an electrical wire is limited by the cross-sectional area of the wire, due to the increase in RC time constant with increased resistance. Large numbers of high-speed electrical signals cannot be effectively multiplexed into a single electrical wire, hence, large numbers of wires must be routed out of the brain. Typically, in high-density multielectrode recording systems, one lithographically defined electrical trace is used per recording site. Creating such complex electrical wiring becomes increasingly difficult for long probe lengths, e.g., with lengths of centimeters.

In order to maintain the advantages of electrodes, single neuron precision based on endogenous neural signals while enabling improved scaling performance, we turn to photonics. Telecommunications has moved from electrical to optical data transmission because of the high bandwidths and low power losses enabled by optics in comparison to electrical conductors;^4^ the same may be helpful for neural readout technologies. Because optical radiation heats brain tissue and scatters off tissue inhomogeneities, a wired (i.e., fiber or waveguide based) optical solution may be desirable, i.e., using optical fibers to guide light so that it need not travel through the tissue itself. Second, to minimize volume displacement, signals from many neurons should be multiplexed into each optical fiber. Third, ideally, the sensing mechanism would rely only on endogenous signals, e.g., electrical or magnetic fields from the firing neurons, rather than imposing a need for exogenously introduced protein or nanoparticle contrast agents. With ∼100,000 neurons per ${\mathrm{mm}}^{3}$ in the cortex, or a median spacing of roughly one neuron per cube of size ${\left(21.5\;\mu m\right)}^{3}$, we require an axial resolution of sensing in the range of tens of micrometers. The system should be compatible with a variety of form factors, e.g., thin flexible fibers suitable for minimally invasive endovascular delivery,^5^^,^^6^ or rigid pillars suitable for direct penetration of the brain parenchyma.^7^

Our proposed architecture is based on two powerful technologies developed by the photonics industry: fiber optic reflectometry, which enables optical fibers to act as distributed sensors,^8^^–^^11^ and electro-optic modulators based on the plasma dispersion effect, which generate large changes in the index of refraction of a waveguide in response to relatively small applied voltages.^12^^–^^16^ By combining reflectometry with electro-optic modulation, we propose that it would be possible to do spatially multiplexed neural recording in a single optical fiber.

## 2 Design Principles

**Fig. 1:**
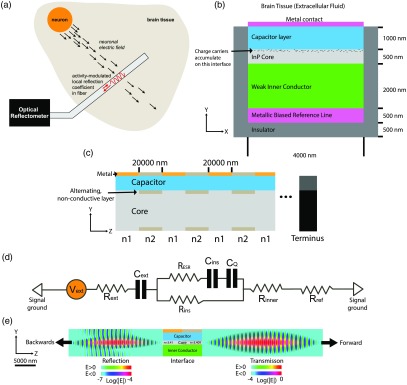
(a) High-level architecture. An optical fiber inserted into the brain acts as a distributed sensor for neuronal activity, which is read out by an optical reflectometer. (b) Axial cross-section of the probe. When a voltage is applied across the capacitor layer, free-charge carriers in the inner conductor and core build up on the surface of the capacitor layer and alter the refractive index in the core. A high capacitance is desired to improve sensitivity. (c) Longitudinal cross-section of the reflectometric probe. Alternating segments of higher and lower refractive index create baseline reflections at their interfaces, the intensities of which are modulated by the local extracellular voltage. The difference between $n_{1}$ and $n_{2}$ is generated by a thin layer of nonconductive material with a different index of refraction, which also serves to localize voltage-dependent refractive index changes to alternating segments. On the surface of the fiber, there are alternating sections of metal contact pads and oxide, to separate sensing and nonsensing regions. (d) Equivalent circuit diagram of the device. The equivalent circuit of the device consists of a resistor representing each of the material layers between the neuron and the metal reference line, and three capacitors, one of which ($C_{\mathrm{ext}}$) represents the interfacial capacitance, one of which ($C_{\mathrm{ins}}$) represents the capacitance of the capacitor layer, and one of which ($C_{Q}$) represents the capacitance due to the non-negligible charge centroid in the semiconducting core. The effective series resistance $R_{\mathrm{ESR}}$ of the insulating region capacitor can be neglected provided the capacitor has high quality factor Q at 1000 Hz, and the parallel resistance of the insulating capacitor layer $R_{\mathrm{ins}}$ can be neglected provided it is much larger than $R_{\mathrm{ref}}$. $R_{\mathrm{ref}}$ is the resistance of the metallic reference line, $R_{\mathrm{inner}}$ is the resistance of the weak inner conductor layer and $R_{\mathrm{ext}}$ is the resistance of the brain–electrode interface. If, in addition, $R_{\mathrm{ref}}$ is chosen to be larger than the other resistances in the circuit, the capacitances $C_{\mathrm{ins}}$, $C_{Q}$, and $C_{\mathrm{ext}}$ may be treated as series capacitances. (e) Optical simulation: We used the MIT Electromagnetic Equation Propagation (MEEP) package to simulate a waveguide with a silicon core divided into two regions. We used Si rather than InP as the simulated core material, because of the availability of well-validated tools for Si electrostatics simulation. In the first region, the core consisted of a 500 nm layer of silicon (n=3.410). In the second region, the core consisted of a 460 nm-wide layer of silicon with two 20 nm layers of a material with n=3.40 both above and below. The effective refractive index in the second region was thus 3.409, corresponding to $\Delta n_{0}=10^{-3}$. The electric field profiles are shown on a logarithmic scale for the waves transmitted (right) and reflected (left) from the boundary between the regions, shortly after the reflection event. The left and right images have been normalized separately. The maximum value in the left image is $∼10^{4}$ times smaller than the maximum value in the right image, consistent with a value of R on the order of $10^{-8}$ for $\Delta n_{0}=10^{-3}$.

Reflectometers are capable of measuring changes in the index of refraction along the length of an optical fiber by sending optical pulses down the length of the fiber and recording the times and magnitudes of returning reflections.^9^ We propose to use reflectometry to sense neural activity at many points along the length of an optical fiber, as shown in Fig. 1(a). The goal is to send a pulse of light into the fiber and to measure the reflections and their timing to determine the one-dimensional profile of neural activity along the length of the fiber. The local voltage at a given position along the fiber will modulate its local index of refraction via the free carrier dispersion effect, giving rise to reflections. A reflectometer located outside the brain would then determine, at each time, the spatial profile of extracellular voltage along the length of the fiber.

### 2.1 Fiber-Optic Reflectometry

To determine the magnitude of the reflections generated by a change in local refractive index inside a fiber, note that when an electromagnetic plane wave propagates in a material with refractive index $n_{1}$ and is normally incident on a material with refractive index $n_{2}$, the power reflected is given by the Fresnel equation:

$$R={\left(\frac{n_{1}-n_{2}}{n_{2}+n_{1}}\right)}^{2}.$$ (1)

The waveguide under consideration will be divided into alternating segments of refractive indices $n_{1}$ and $n_{2}$, respectively [Fig. 1(c)]. We define $\overset{¯}{n}=(n_{1}+n_{2})/2$ and $\Delta n_{0}=n_{2}-n_{1}$. At every interface between the two segments, a reflection is generated of magnitude:

$$R_{0}={\left(\frac{\Delta n_{0}}{2\overset{¯}{n}}\right)}^{2}.$$ (2)

FDTD simulations^17^ of the waveguide structure using the MEEP^18^ software package [Fig. 1(e)] confirm that the baseline reflections are of the predicted order of magnitude per this simple model.

Assuming now that an event (i.e., local neural activity) causes $n_{2}$ to increase by a small amount $\Delta n\ll \Delta n_{0}$, the resulting reflections generated by the interface are given by

$$R={\left(\frac{\Delta n_{0}+\Delta n}{2\overset{¯}{n}}\right)}^{2}=R_{0}+\frac{\Delta n_{0}\Delta n}{2{\overset{¯}{n}}^{2}}+O(\Delta n^{2}).$$ (3)

The change in the reflections generated at the interface due to the event is thus

$$\Delta R=\frac{\Delta n_{0}\Delta n}{2{\bar{n}}^{2}}.$$ (4)

#### 2.1.1 Resolution of reflectometry

*Reflection intensity sensing*. Reflectometers are limited both in the minimum value of R that they can sense (termed the sensitivity) and in the minimum value of ΔR that they can sense (termed the resolution). In our device, the baseline power reflected at the boundaries between $n_{1}$ and $n_{2}$ will be much greater than the sensitivity of the reflectometer. Thus, the ability of the reflectometer to measure a change in the index of refraction is limited by its resolution, which is in turn fundamentally limited by photon shot noise. For the simple case of a time-domain reflectometer, the number of photons registered at the detector due to a reflector of magnitude $R_{0}$ is given by

$$N_{\text{reflected}}=\mathrm{QE}\cdot \frac{P}{h\cdot c/\lambda }\cdot R_{0}\cdot \frac{1}{\mathrm{BW}},$$ (5)

where P is the power entering the fiber, QE is the detector quantum efficiency, and BW is the sensing bandwidth. With a signal-to-noise ratio of $N/\sqrt{N}$ due to photon shot noise, the resolution of the detector is given in dB by

$$dB_{\text{shot noise limit}}=10\log_{10}\left(1+\frac{1}{\sqrt{N}}\right)=10\log_{10}(1+\frac{1}{\sqrt{\mathrm{QE}\cdot \frac{P}{h\cdot c/\lambda }\cdot R_{0}\cdot \frac{1}{\mathrm{BW}}}}).$$ (6)

In all that follows, we will assume a bandwidth of 1 kHz, a quantum efficiency of 1, and a free-space wavelength λ=1550  nm. Note that a higher bandwidth would be required to see the detailed shapes of individual action potentials, as may be required for spike sorting. For P=100  mW, QE=1, BW=1  kHz, and $\Delta n_{0}=10^{-4}$, corresponding to $R_{0}=2.16\times 10^{-10}$ (−96.6  dB), we then have a shot noise limited resolution of 0.01 dB, similar to existing reflectometers. More generally, for a signal ΔR to be sensed on top of a signal $R_{0}$, we must have

$$10\log_{10}(1+\frac{\Delta R}{R_{0}})\geq 10\log_{10}(1+\sqrt{\frac{\mathrm{BW}}{\mathrm{QE}\cdot \frac{P}{h\cdot c/\lambda }\cdot R_{0}}}).$$ (7)

As we describe below, the device will be sensitive to changes in the index of refraction on the order of $\Delta n∼10^{-7}$ to $10^{-6}$. Thus, because $\Delta n_{0}\gg \Delta n$, the device operates in the linear regime of Eq. (3), so Eq. (7) may be conveniently re-expressed as

$$\frac{\Delta n}{\bar{n}}\geq \sqrt{\frac{\mathrm{BW}}{\mathrm{QE}\cdot \frac{P}{h\cdot c/\lambda }}}.$$ (8)

**Fig. 2:**
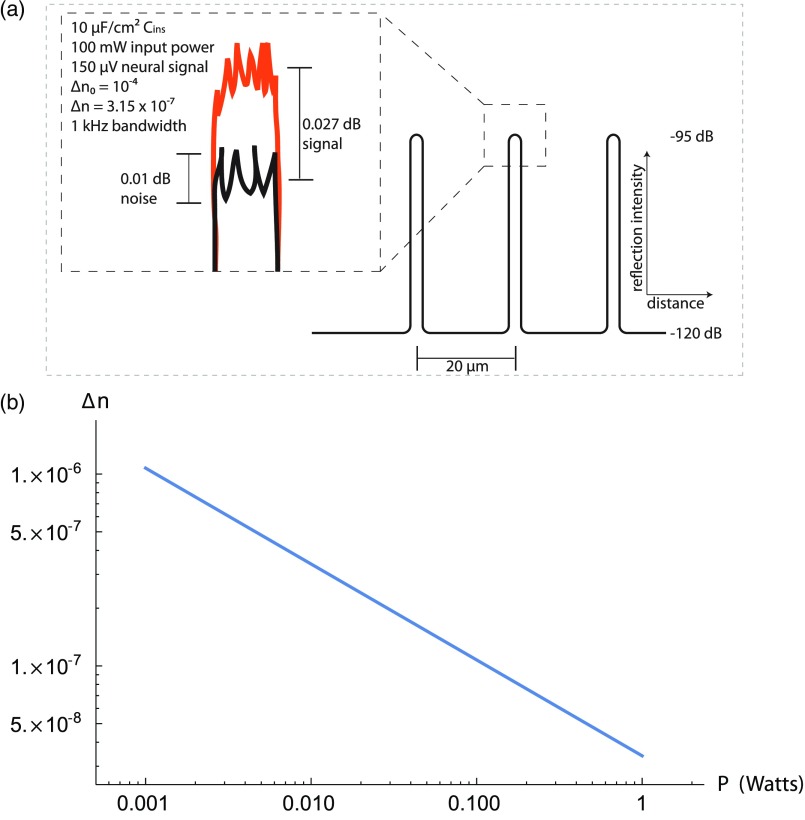
Minimum resolvable value of Δn. (a) A schematic example of the expected output trace. Black color is the baseline reflection registered by the device; orange color is the reflection measured when the neuron fires. Spatial resolution is exaggerated for illustration. (b) The minimum change in the index of refraction of the optical fiber that can be sensed by an ideal, shot noise-limited reflectometer is shown as a function of the laser power P for a 1-kHz bandwidth, index of refraction of 3.36, quantum efficiency close to 1, and 1550-nm wavelength.

With the choices of the bandwidth, quantum efficiency, and wavelength given above, the resolution limit is strictly a function of power. The minimum resolvable Δn is shown as a function of P in Fig. 2. Notably, Eq. (8) is independent of $\Delta n_{0}$ in the linear regime. The inset in Fig. 2 shows a schematic example of the expected output.

So far, we have discussed the detection of changes in the refractive index via the modulation of reflectivity at each interface. An alternative strategy to detecting changes in the refractive index that accompany voltage signals is to measure the phase of the reflected light. This phase measurement can be performed with the identical Fourier-domain reflectometry scheme as for the amplitude-based measurement.

*Spatial resolution*. Other noise sources will also impact resolution in a realistic case, including laser power or phase noise and photodetector/amplifier/ADC noise. In particular, optical phase noise associated with the laser is limiting in current optical frequency-domain reflectometry (OFDR) systems;^19^ Littman–Metcalf external cavity tunable lasers, with narrow linewidths and low phase noise, can be swept at 1-kHz repetition rates over an optical frequency range of several THz, leading to an OFDR “spatial” resolution of roughly 20  μm, which conveniently aligns with the average spacing between neurons in the cortex.

*Repetition rate*. Current commercial reflectometers achieve roughly 12-Hz repetition rates over 8.5 m. This corresponds to a measurement time of 1 ms for any given 10 cm segment of fiber, so using a similar device we anticipate that it would be possible to sense reflections along the length of a 10-cm fiber with a repetition rate of 1 kHz using frequency-domain reflectometers. In an OFDR system, the scan rate is limited by the frequency of laser wavelength scanning, the range of the scan determines the resolution, and the wavelength resolution of the scan and of the detector determines the scan range. Swept-source OCT constitutes demonstration of swept-source interferometry at a bandwidth of many kHz.^20^

### 2.2 Electro-Optic Modulation

Silicon electro-optic modulators are widely used in photonics to alter the propagation of light through a material in response to an applied voltage.^15^^,^^21^ Typical applications of electro-optic modulators take the form of electrically controlled optical switches: signals of roughly 5 V are used to drive optical phase shifts on the order of π. These devices are optimized for GHz bandwidths, with the goal of providing high speed, low power microchip interconnects,^14^ with bandwidths up to 30 GHz possible.^22^ Here, however, we are interested in the application of similar device physics to a very different problem: sensing extracellular neuronal voltages on the order of 100  μV at 1 kHz rates. Thus, our required switching rate is 1 millionfold slower, yet our required electrical sensitivity is on the order of 1 millionfold better. We are thus concerned with the design of electro-optic modulators optimized for sensitivity rather than bandwidth.

#### 2.2.1 Free carrier dispersion effect

The design shown in Fig. 1(b) consists of an extended multilayer semiconductor waveguide on a biased metal substrate, surrounded on three sides by insulation and on the fourth side by brain tissue or extracellular fluid. The “inner conductor” and “core” layers are weak, transparent conductors which function as resistive layers between the brain and the biased reference line. Throughout, we will assume that the core is made of n-doped InP, due to its large free-carrier dispersion effect,^23^ although other core materials are possible (see Sec. 4). Both above and below the core, there are ∼5  nm thick layers [Fig. 1(c)] in which the material alternates along the length of the fiber between the core material and a nonconductive material. The nonconductive material is chosen to have a refractive index that differs from that of the InP core by 0.01. At the boundaries between the alternating regions, there is an effective change in the index of refraction of $\Delta n_{0}∼10^{-4}$, giving rise to a reflection $R_{0}∼2.16\times 10^{-10}$ as per Eq. (2). This value of $R_{0}$ is chosen to avoid significant attenuation over the length of the fiber. Note that the sensitivity is independent of $\Delta n_{0}$ as long as we remain in the linear regime of Eq. (3). The alternating regions are 20  μm in length, with randomness introduced on the order of 1  μm to avoid the formation of strong peaks in the reflectivity with wavelength due to interference. The effective spatial resolution in this design is then limited by the linear density of sensing sites, which are spaced at 40  μm from center to center, rather than by the underlying 20-μm spatial resolution of the reflectometer.

Above the core, there is an insulating layer that serves both as cladding, and as a capacitor over which most of the voltage will drop. The capacitor layer must be thick enough to serve as effective optical cladding, while also having a high capacitance. To satisfy these constraints, a material like barium titanate, strontium titanate, or calcium copper titanate may be preferred. We set this layer’s thickness to ∼1  μm. Clearly, the titanate layer must have lower refractive index than the core to act as a cladding. Although the optical properties of the titanate layer depend on its preparation, the band gap of a single crystal of barium titanate occurs at 3.2 eV,^24^ and the refractive index of barium titanate is ∼2.4 for λ=600  nm,^25^ so it is safe to assume n<2.4 for λ=1.5  μm. Above the capacitor layer, there are alternating regions of metal and insulator, with the insulating regions coinciding with the alternating layers in the waveguide core. The metal regions provide the electrical interface to the brain and serve to define the sensing locations.

The InP core and inner conductor are doped and biased appropriately to allow most of the voltage to drop over the capacitor layer while maintaining low levels of optical attenuation, for example, $∼10^{17}\;{\mathrm{cm}}^{-3}$. Other major materials requirements on the inner conductor are that it should ideally form an ohmic contact with both the InP core and the metal reference layer, and that its refractive index needs to be smaller than that of the n-doped InP, which is around 3.17 at λ=1.5  μm. Potential materials candidates then include type III-V semiconductors with lower refractive indices, such as GaP, or II-VI semiconductors, such as ZnSe or CdS. These have lower refractive indices at 3.05, 2.45, and 2.30, respectively. These can be expitaxially grown on InP or vice versa due to the small lattice mismatch,^26^ and their conductivities can be tuned by doping. On the other hand, it would be important to prevent the formation of a rectifying junction at the semiconductor–semiconductor interface, the existence of which would depend on the band mismatch and doping levels. It might be possible to lower the junction barrier by, for example, minimizing the band gap difference between the two adjacent semiconductors. In the below analysis, we will assume that all junctions can be made ohmic. Note that the inner conductor is chosen to be thick enough to prevent optical attenuation due to the metal substrate (although there are other possible methods to reduce attenuation due to the metal, e.g., by removing the metal from the region directly under the waveguide, as in Ref. 13), and the metal substrate is chosen thick enough to provide a high-fidelity biased reference throughout the fiber.

The design relies on the free-carrier dispersion effect (also known as the plasma dispersion effect): the index of refraction within the InP core changes due to the accumulation of charge carriers in the InP when a voltage is applied across the capacitor layer.^13^^,^^23^^,^^27^ Many current integrated semiconductor electro-optic modulators are based on the free-carrier dispersion effect.^12^^,^^13^ In addition to the free-carrier effect, there exist other modalities of electro-optic modulation, such as the linear electro-optic (Pockels) effect,^28^ the quadratic electro-optic effect (Kerr)^29^ and the Stark effect.^30^ All of these effects would benefit from reducing the thickness d of the insulator layer to create a large electric field V/d.^31^ However, the free-carrier effect uniquely depends on the “charge,” rather than the field, and can thus be amplified further by increasing the relative permittivity of the capacitor layer. In short, we need a large capacitor, which can be achieved by reducing the thickness and increasing the relative permittivity. For a material with a suitably large value of $\varepsilon_{r}/d$, the change in refractive index due to the free-carrier effect will be much larger than the changes that can be obtained via the other electro-optic effects. Although we focus on the free-carrier effect here, it should be noted that novel electro-optic materials, such as potassium tantalate niobate,^32^ with extremely high electrooptic coefficients compared to standard electrooptic materials like lithium niobate, could also potentially make possible designs based on the Pockels or Kerr effects.

An appropriate bias voltage will be applied through the reference conductor to ensure that the InP core layer operates in accumulation. This is necessary in order to avoid depletion,^33^ which would reduce the charge recruited to the surface of the capacitor for a given change in extracellular voltage, and thus reduce the sensitivity. Thus, we use the reference potential in the brain plus some fixed bias to achieve accumulation in the InP core along the waveguide. If needed, this bias could be achieved locally, but as long as the brain has no large voltage differences (e.g., >1  V), one global bias may be sufficient to allow the entire InP core to operate in accumulation.

Changes in the index of refraction in the free-carrier modulated region of the InP may be modeled as changes in the overall effective index of refraction of the fiber.^34^ The magnitude of this effective change is given by weighting the magnitude of the change in the free-carrier modulated layer by the percentage of power contained in that layer, i.e.,

$$\Delta n_{\mathrm{eff}}=(1-\eta )\Delta n_{\text{active}},$$ (9)

where $n_{\text{active}}$ is the index of refraction in the free-carrier modulated layer and 1−η is the fraction of the power in the beam contained in the active region.

We will denote by d the thickness of the capacitor layer, by b the thickness of the layer of injected charge carriers in the InP, and by a the remaining thickness of the InP layer. An order-of-magnitude approximation for η is then given by

$$\eta ≅\frac{a}{\left(a+b\right)},$$ (10)

and we have

$$\Delta n_{\mathrm{eff}}=[1-\frac{a}{\left(a+b\right)}]\Delta n_{\text{active}}.$$ (11)

For this reason, the InP waveguide is chosen to be thin to maximize the percentage of the optical wave contained in the layer containing the injected charges. Because of the deep subwavelength thickness of the active layer, a precise calculation of $\Delta n_{\mathrm{eff}}$ could be done using a full-vectorial Maxwell simulation of the waveguide modes,^13^ but for our purposes, the approximation of Eq. (11) suffices to illustrate the basic scaling.

Upon applying a voltage across the capacitor layer, the density of charge carriers injected into the active layer inside the InP core, denoted by ΔQ, is simply given by the equation for a parallel plate capacitor:

$$\Delta Q=\frac{C_{\mathrm{ins}}}{eAb}\Delta V_{\mathrm{ins}},$$ (12)

where $C_{\mathrm{ins}}/A$ is the capacitance per unit area of the insulator, e is the electron charge, b is the thickness of the layer of injected charge carriers in the InP, and $\Delta V_{\mathrm{ins}}$ is the voltage dropped over the insulating region. Equation (12) may be recast in terms of the total voltage ΔV applied over the device by introducing an effective capacitance $C_{\mathrm{eff}}$, such that

$$\Delta Q=\frac{C_{\mathrm{eff}}}{eAb}\Delta V.$$ (13)

In practice, $C_{\mathrm{eff}}$ will only deviate significantly from $C_{\mathrm{ins}}$ when the capacitance of the brain-fiber interface is significant (discussed below). The change in refractive index in the region with the injected charge is related to the change in the carrier concentration by a power law.^23^ When the injected carriers are electrons, the magnitude of the electro-optic effect in InP is greatest. The relation for the change in refractive index in the injected charge region is then

$$\Delta n_{\text{active},h}=C_{e}\frac{C_{\mathrm{eff}}}{eAb}\Delta V,$$ (14)

where $C_{e}$ is an empirically defined constant.

For InP, the value of $C_{e}$ is given for 1.55  μm light by^35^

$$C_{e}=-5.6\times 10^{-21}\;{\mathrm{cm}}^{3}.$$ (15)

This wavelength is chosen because the waveguide is made of InP, and InP is transparent at these telecom wavelengths. Telecom windows are around 1.3 and 1.5  μm due to local minima of the absorption of water, a hard-to-avoid contaminant in silica fibers. The exact choice of wavelength is not critical to the sensing mechanism itself; according to the Drude model of the free-carrier dispersion effect,^35^ the coefficient in Eq. (12) is quadratic in the wavelength.

Similar values are obtained for other semiconductors and other wavelengths.^23^^,^^35^ To find the effective refractive index within the InP waveguide, we multiply Eq. (14) by the volume factor 1−η from Eq. (10). Assuming b≪a (i.e., that the injected charge layer is deeply subwavelength while the waveguide core thickness is on the same order as the wavelength), we find

$$\Delta n_{\mathrm{eff}}≅C_{e}\frac{1}{a+b}[\frac{C_{\mathrm{eff}}}{eA}\Delta V].$$ (16)

Note that for a given waveguide thickness (i.e., a+b constant), the result is independent of the thickness of the charged layer b. We will henceforth take a+b∼500  nm. For a value of $C_{\mathrm{eff}}/A$ on the order of $10\;\mu F\;{\mathrm{cm}}^{-2}$, justified below, we find $\Delta n_{\mathrm{eff}}∼2\times 10^{-7}$ for ΔV∼100  μV.

#### 2.2.2 Effects of other capacitances

The brain–electrode interface also has a capacitance $C_{\mathrm{ext}}$ of $∼100\;\mu F\;{\mathrm{cm}}^{-2}$,^36^^,^^37^ which arises due to the presence of an electrical double-layer [see Fig. 1(d)].

In addition, there is a capacitance $C_{Q}$ due to the finite length scale of the charge distribution inside the semiconductor. This latter capacitance is given by $C_{Q}=\varepsilon_{\text{core}}\varepsilon_{0}A/d_{Q}$, where $\varepsilon_{\text{core}}$ is the relative permittivity of the core material, $\varepsilon_{0}$ is the permittivity of free space, A is the area of the sensing region, and $d_{Q}$ is the charge centroid. The core can be one of many semiconductor materials (e.g., Si, InP), leading to similar fundamental electrostatics. We performed semiconductor simulations using the Sentaurus TCAD device simulator (version K-2015.06, June 2015) to evaluate $C_{Q}$. We used Si as the simulated core material, because of the availability of accurate and readily available tools for Si electrostatics simulation. To calculate the charge centroid $d_{Q}$, we simulated the electrostatics of an interface between silicon n-doped to a level $10^{17}\;{\mathrm{cm}}^{-3}$ and a layer of oxide with relative permittivity of $\varepsilon_{r}=5$ and thickness of d=1  nm. When the silicon was in accumulation, the charge centroid was found to be 2.4 nm. The charge centroid is expected to be similar for our setup provided the value of $\varepsilon_{r}/d$ is similar for the capacitor layer, which it would be for a layer of barium titanate with $\varepsilon_{r}=5000$ and d=1000  nm (see below). It is more difficult to do these simulations for less common materials like InP, but we anticipate that the charge centroid for InP will be similar. Thus, in all following calculations, we will assume a capacitance of $4.5\;\mu F\;{\mathrm{cm}}^{-2}$ for the interface between the core and capacitor layer.

The electrostatics simulations also showed that, although the total charge ΔQ recruited to the capacitor layer surface upon application of a voltage is greater for higher doping levels, the relative change in charge (ΔQ/Q) is greater for lower doping levels. However, for ΔQ≪Q, the sensitivity condition in Eq. (8) depends to a good approximation only on ΔQ, not on ΔQ/Q, so the sensitivity of the device is increased for higher doping levels.

Figure 1(d) shows an equivalent circuit diagram of the device, which includes the interfacial capacitance and the capacitance associated with the charge distribution. At <1000  Hz, and subject to appropriate materials choices (see Sec. 2.2.3), the impedance of the circuit is dominated by these three capacitors rather than by purely resistive elements of the circuit. For this reason, we may ignore the purely resistive elements and treat the capacitances as though they were in series. To a good approximation, therefore, the charge that accumulates on the surface of the insulating region in response to a voltage ΔV across the entire device is given by

$$Q=C_{\mathrm{eff}}\Delta V,$$ (17)

where the effective capacitance $C_{\mathrm{eff}}$ of the surface and insulating region capacitors in series is

$$C_{\mathrm{eff}}=\frac{1}{\frac{1}{C_{\mathrm{ins}}}+\frac{1}{C_{\mathrm{ext}}}+\frac{1}{C_{Q}}}.$$ (18)

The capacitance of the insulating region is given by

$$C_{\mathrm{ins}}=\epsilon_{0}A\frac{\epsilon_{r}}{d},$$ (19)

where d is the thickness of the insulating region, $\varepsilon_{r}$ is the relative permittivity, A is the area of the sensing region, and $\varepsilon_{0}$ is the permittivity of free space. Along with the laser power discussed above, the capacitance per unit area of the capacitor layer, $C_{\mathrm{ins}}/A$, will be the primary figure of merit for determining the sensitivity and noise characteristics of the device.

**Fig. 3:**
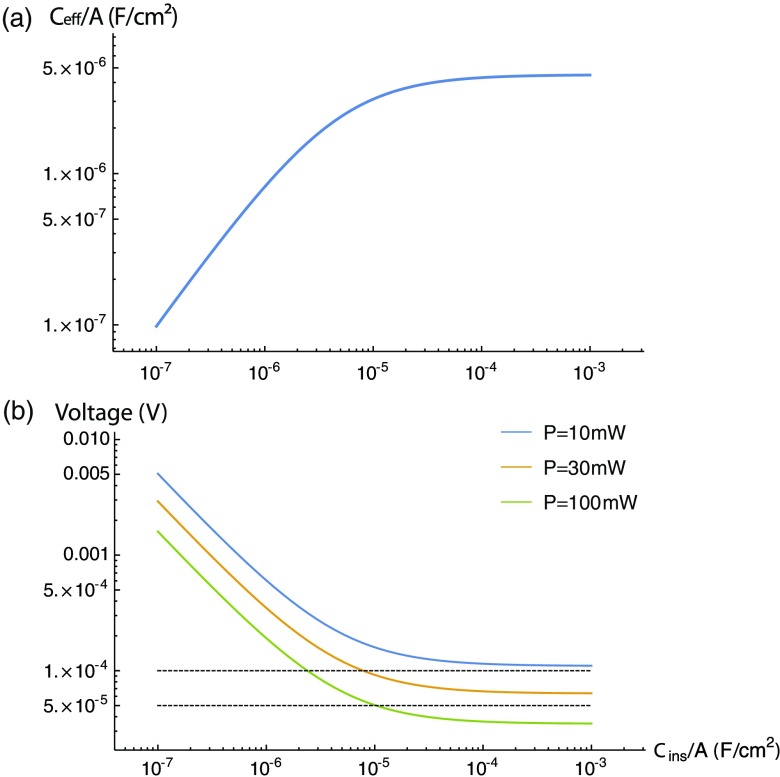
Properties of the design parametrized by $C_{\mathrm{ins}}/A$. (a) The effective capacitance $C_{\mathrm{eff}}/A$ given in Eq. (18) is shown as a function of $C_{\mathrm{ins}}/A$, assuming a capacitance of $100\;\mu F\;{\mathrm{cm}}^{-2}$ at the surface of the device and a capacitance of $4.5\;\mu F\;{\mathrm{cm}}^{-2}$ at the interface between the core and capacitor layers. (b) The minimum detectable change in voltage [obtained from Eqs. (8) and (16)] is shown as a function of $C_{\mathrm{ins}}/A$ for systems with (from top to bottom) a 10-mW laser (blue), a 30-mW laser (orange), and a 100-mW laser (green). The black dashed lines correspond to 50 and 100  μV. To sense signals at the 50  μV level with a 100-mW laser, a capacitance on the order of $10\;\mu F\;{\mathrm{cm}}^{-2}$ is necessary.

The effective capacitance $C_{\mathrm{eff}}$ is shown in Fig. 3(a) as a function of the capacitance $C_{\mathrm{ins}}$ of the capacitor layer, assuming a surface capacitance per unit area^36^^,^^37^ of $∼100\;\mu F\;{\mathrm{cm}}^{-2}$ and a sensing length of 20  μm. Note that the effective capacitance ceases to increase for values of $C_{\mathrm{ins}}/A\gg 4.5\;\mu F\;{\mathrm{cm}}^{-2}$, because for these values, the capacitance is dominated by the core-capacitor interface.

#### 2.2.3 Noise sources

A primary electrical constraint on the device is that the impedance at 1 kHz must be dominated by the capacitor layer. If the effective capacitance per unit area of the capacitor is $C_{\mathrm{eff}}=5\;\mu F\;{\mathrm{cm}}^{-2}$, corresponding to a value $C_{\mathrm{ins}}=10\;\mu F\;{\mathrm{cm}}^{-2}$, then the capacitance of a region with width 4  μm and length 20  μm is 4 pF, corresponding to an impedance of 40  MΩ at 1 kHz.

Assuming that the metal layer has a resistivity no greater than 100  nΩ m (10× that of silver), if the metal layer is made at least 500 nm thick, it will have a resistance of 10,000Ω along the entire length of the fiber. The resistance of the inner conductor and core will be negligible compared to the huge capacitive impedance, provided they are chosen to be semiconductors. Finally, we must consider the voltage noise on the recording site itself, i.e., the metal contact pad interfacing directly with the brain. The recording site is often modeled as a constant phase element^38^ and noise contributions come from the real part of its impedance,^2^ and are frequency dependent. We choose to write it in terms of parameters G and m, with an impedance of $Z_{e}=1/G{\left(j\omega \right)}^{-m}$. The parameter G reflects the conductivity of the material, and the parameter m is often related to surface roughness and transport to the metal–electrolyte interface,^39^ with typical parameters ranging from 0.5 to 0.9. We will assume m=0.5 for representing a rough surface, and a 1 kHz impedance magnitude of 0.1  MΩ. Thus, the resistance of the device is dominated by the recording site, as opposed to the ground lead or other elements, and the above parameters amount to a total RMS noise over a 1 kHz band, found by integrating 4kBTRe(Ze)df from f=0  Hz to f=1000  Hz, of ∼1  μV. This model agrees with what is found experimentally for similar sized electrode pads.^39^

Efforts to reduce the recording site impedance are only needed for adjusting the noise influence of the recording site itself. Even an unplated gold surface will be sufficient here, because instead of 0.1  MΩ for an electroplated surface, we will have Re(Z)=1  MΩ, with a resulting noise of ∼4  μV RMS instead of the 1  μV RMS calculated above. Only if $C_{\mathrm{eff}}$ was increased dramatically (e.g., to the equivalent impedance of ∼1  MΩ at 1 kHz), would efforts be needed to reduce the recording site impedance to prevent attenuation of the signal via the voltage divider. In any case, the voltage drops primarily over the capacitor layer and is not attenuated by resistors prior to the capacitor, and these electronic noise voltages are lower than the sensitivity of the device, which is limited by optical shot noise, and so can be neglected. Note that the impedances given here are also large enough for the input impedance of an implanted recording device.^40^

Other forms of exogenous noise include mechanical bending of the fiber and thermo-optic effects, which may be particularly significant given the small width of the waveguide. However, these effects are expected to occur at a much lower frequency than the ∼1  kHz frequency content of spikes, and thus can be filtered out. Likewise, static or slowly changing bends (e.g., due to the heart beat) in the fiber can be subtracted off.

#### 2.2.4 Dynamic range

Local field potentials in the brain may vary by up to hundreds of millivolts, generating fields on the order of $1\;{\mathrm{kV}\;\mathrm{cm}}^{-1}$ across a 1-μm capacitor layer. By contrast, the dielectric breakdown strength of barium titanate is roughly $10\;{\mathrm{kV}\;\mathrm{cm}}^{-1}$, so dielectric breakdown is unlikely to be an issue.^41^ On the other hand, the dynamic range of the device may be limited by the density of states in the core, and thus it will be necessary to adjust the bias of the device (using the conductive reference layer) in order to ensure that the device can function in accumulation. If the device is allowed to function in depletion, $C_{Q}$ will be much smaller than $C_{\mathrm{ins}}$, thus reducing the sensitivity. Similarly, operation in inversion will suffer from deep depletion effects.

#### 2.2.5 Tissue heating

When we send light down the fiber, some light power may dissipate into the tissue. Depending on the level of round-trip light attenuation in the waveguide, each probe will dissipate a fraction f of the applied light power P. We next evaluate the acceptable level of such dissipation and how this constrains the device properties.

The human brain endogenously dissipates 25 W or $19\;\mathrm{mW}\;{\mathrm{mL}}^{-1}$. The blood perfusion rate^42^ of human brain gray matter and white matter is roughly $r_{\text{perfusion}}=35000\;{\mathrm{Wm}}^{-3}\;{}^{\circ}C^{-1}$. To avoid >2°C brain temperature rise, per the requirements laid out in Ref. 3 and elsewhere, we then require that each probe is surrounded by a perfusion volume of $V_{\text{perfusion}}\approx \frac{f\cdot P}{r_{\text{perfusion}}\cdot 2{}^{\circ}C}$.

For a sense of scale, assuming that a 100-mW laser is used for the reflectometer, if f≈50% of this light power is dissipated into the tissue on a round-trip reflection, we then require a 250  μL perfusion volume, or a cylinder of radius 1.5 mm around each probe, assuming a 10-cm probe length. An attenuation of 50% over 20 cm corresponds to ∼3  dB over the length of the fiber, or $∼0.15\;\mathrm{dB}\;{\mathrm{cm}}^{-1}$, on the order of the intrinsic optical attenuation of silicon^43^ or indium phosphide.^44^ An additional potential source of tissue heating arises from transverse scattering of light at the interfaces between the successive waveguide segments of different refractive index. Using MEEP simulations to quantify the amount of light scattered out of the waveguide core, for adjacent segments with refractive indices of 3.409 and 3.41, we estimate that there will be a $3\times 10^{-5}\%$ loss per boundary. With 500 boundaries per centimeter, this means a 0.015% loss per centimeter or 0.3% loss over a round trip in a 10-cm fiber. However, if $\Delta n_{0}$ is $∼10^{-4}$ instead, as discussed above, the amount of scattering generated this way is expected to be substantially reduced.

Attenuation due to bending is expected to be insignificant, with silicon-on-insulator waveguides reported to experience attenuation of only ∼0.1  dB per 90 deg turn at a radius of curvature of 1  μm. Finally, to avoid transmitting any light into the brain tissue itself, a strong reflector can be placed at the end of the probe. Because the reflectometer has high spatial resolution, a large reflection from the end of the probe is not expected to interfere with the measurements.

## 3 Material Selection for the Capacitor Layer

The key figure of merit determining the properties of the device is the capacitance per unit area of the capacitor layer, $C_{\mathrm{ins}}/A$. Along with the laser power, the figure of merit determines the sensitivity via Eq. (16). In Fig. 3(b), the sensitivity of the device is shown as a function of $C_{\mathrm{ins}}/A$. The vertical axis shows the minimum voltage signal that can be resolved using a shot noise-limited reflectometer, as calculated using Eqs. (8) and (16). The power law region (a straight line on the log–log plot) corresponds to the region in which $C_{\mathrm{eff}}\approx C_{\mathrm{ins}}$, so that the reflection coefficient $R\propto C_{\mathrm{ins}}\Delta V/A$. For values of $C_{\mathrm{ins}}/A$ much greater than $4.5\;\mu F\;{\mathrm{cm}}^{-2}$, we have $C_{\mathrm{eff}}\approx C_{Q}$, so the sensitivity does not improve with increasing $C_{\mathrm{ins}}/A$.

Materials such as barium titanate, strontium titanate, and calcium copper titanate would likely be able to achieve a sufficiently large value of $C_{\mathrm{ins}}/A$ while also separating the core from the metal sensing pads. The chosen material must be able to maintain its high relative permittivity while film thickness is scaled down sufficiently to enable a high capacitance. Since dielectric properties often arise from grain boundaries within the material, the achievable grain size sets an approximate lower bound on the film thickness that can be utilized. Barium titanate films have been demonstrated with relative permittivities of roughly 5000 with grain sizes around 1  μm,^45^ or with relative permittivities of 2500 with grain sizes of 100 nm.^46^ Likewise, calcium copper titanate ceramics have been fabricated with relative permittivities between 1000 and 10,000 and grain sizes from hundreds of nanometers to micrometers.^47^ Finally, relative permittivities on the order of $10^{5}$ seem to be possible with larger grain sizes.^48^^,^^49^

We are not aware, however, of direct measurements of the dielectric properties of high-dielectric ceramics in films of <1  μm thickness grown in InP substrates, thus verification of these properties should be a key question for early experimental studies of voltage probes like the one proposed here. A further potential concern with using dielectrics, such as barium titanate, is the presence of hysteresis in such materials.^50^ Since the neuronal signals involve potential changes on the order of 100  μV, the hysteresis is expected to be small, but a detailed experimental characterization would be required.

We will assume that it is possible to fabricate a dielectric film with thickness d∼1μm, and with $\varepsilon_{r}/d∼10^{10}$, for example, a 1  μm-thick film of calcium copper titanate with $\varepsilon_{r}∼10^{4}$, corresponding to a value of $C_{\mathrm{ins}}/A$ of $∼10\;\mu F\;{\mathrm{cm}}^{-2}$. With such a capacitor, the device with a 30-mW laser would be capable of measuring signals at the 100-μV level and the device with a 100-mW laser would be capable of measuring signals at the 50-μV level.

## 4 Discussion

Ultra-large-scale neural recording is highly constrained both by physics and by the biology of the brain.^3^ Here, we have argued that an architecture for scalable neural recording could combine (1) the use of optical rather than electronic signal transmission to maximize bandwidth, (2) confined rather than free-space optics to reduce the effects of light scattering and absorption in the tissue, (3) spatial or wavelength multiplexing within each optical fiber in order to minimize total tissue volume displacement, (4) a thin form factor to enable potential deployment of fibers via the cerebral vasculature, and (5) direct electrical sensing to remove the need for exogenous dyes or for genetically encoded contrast agents.

Traditional electrode-based recording systems require a separate electrical connection for every recording site. They are limited in the depth they can access, because the magnitude of the thermal noise increases with the length of the probe. Furthermore, each connection must be accessed separately by the acquisition system.^51^ By contrast, the architecture proposed here offers several benefits, including the ability to read out neural activity over many centimeters with high sensitivity, the ability to multiplex tens of thousands of recordings into a single fiber with a simplified acquisition system, and the ability to scale the physical dimensions of the fiber without sacrificing performance.

In our proposed design, the 100-μV scale extracellular voltage resulting from a neuronal spike is applied across a thin, high-dielectric capacitor. Charging of the capacitor results in modulating the accumulation layer in the neighboring InP waveguide core, altering the local refractive index of the InP and causing a detectable optical reflection. Reflectometry then enables multiplexed readout of these spike-induced reflections. Notably, the entire design fits into a package with a cross section that is in principle <5  μm on a side (although additional material could of course be added for mechanical support if desired).

Every neuron in a mammalian brain is within a few tens of microns of the nearest capillary,^52^ well within the distance necessary for direct electrical sensing of the action potential,^3^ thus, in principle, the fine microvessels of the cerebral vasculature could serve as a delivery route for neural activity sensors, if the fibers could be made sufficiently thin,^6^ i.e, well below 10  μm for the smallest capillaries. Thus, multiplexing thousands of neural signals into a single optical “wire” of <10  μm thickness could potentially be enabling for novel endovascular approaches to neural interfacing.

It is worthwhile to contrast the proposed system to both microelectrode-based recording and optical imaging solutions. In our design, signals are captured electrically, similar to the recording mechanism of a microelectrode, and then are transduced to an optical communication channel for extracting the data from the brain. By contrast, in imaging approaches, the neuronal signal is transduced into the photochemical state of an indicator dye or protein inside the neuron itself, and then the signal is extracted by irradiating the brain and then capturing emitted fluorescent photons on a camera. Consequently, imaging approaches flood the brain tissue itself with light power and transduce signals via chemicals delivered to the neurons themselves. Our proposed method, in contrast, does not require flooding the brain tissue itself with light: the electrical pickup of the signal does not require power nor exogenous chemical probes, and the data collection is photon-efficient since, to the greatest extent possible, our design confines all light to the inside of the waveguide itself.

A key challenge in implementing such a design is to achieve a figure of merit $C_{\mathrm{ins}}/A$ for the capacitor sufficiently large to allow sensitivity to the neural signals of interest. We think that it would be possible using barium titanate or calcium copper titanate to achieve a figure of merit on the order of $10\;\mu F\;{\mathrm{cm}}^{-2}$, which would allow the device presented here to sense signals of approximately 50  μV with a 100-mW laser.

In addition, supercapacitors with submicrometer thickness can be fabricated that achieve specific capacitances on the order of $1\;\mathrm{mF}\;{\mathrm{cm}}^{-2}$,^53^ which would allow for the detection of 30  μV signals with a 100-mW laser, if they could be made compatible with our device. The sensitivity would also be improved substantially if a core material could be found with a smaller charge centroid. Early experimental studies building on our theoretical estimates should seek to verify that a sufficiently high capacitance can be achieved in the desired form factor.

Several alternative strategies exist for improving the sensitivity of the device. The device senses the voltage in each sensing region twice, at the front and back ends of each sensing region, which could be factored into the analysis to improve SNR. If tissue-heating concerns can be overcome, the sensitivity of the device can be improved by increasing the strength of the laser. The sensitivity can also be increased by using a different core material with a stronger free-carrier dispersion effect. For example, at λ=1.3  μm, there is a maximum in the free-carrier dispersion effect of InP at a doping concentration around $3\times 10^{17}\;{\mathrm{cm}}^{-3}$.^35^ By using a core material with a higher bandgap, such as GaP, it would be possible to perform reflectometry using visible light, for example, around 600 nm, which would increase the sensitivity of the device by increasing the overlap of the optical electric field with the charge-containing region of the core. Alternatively, silicon is also possible as a core material, for simplicity of fabrication. However, it would be necessary for the chosen core material also to have acceptable levels of field-induced birefringence and nonlinear response, effects which could cause frequency conversion or interfere with the reflectometry process. These processes should be evaluated empirically for a given power level, materials choice, and waveguide configuration. Finally, the sensitivity of the proposed device is dependent on the signal to noise ratio of the reflectometer. Although we have applied a conservative estimate of the shot-noise-limited resolution, other sources of noise will have to be minimized to achieve sufficient sensitivity for neural recording.

Finally, a major challenge will be the achievement of an attenuation level low enough to avoid excessive heating of the tissue. The heat dissipation can be reduced by reducing the laser power or using a core material with lower optical attenuation. For this reason, GaP is also an appealing option for the waveguide core, as it has been reported to have intrinsic optical attenuation much less than $0.1\;\mathrm{dB}\;{\mathrm{cm}}^{-1}$ at 600 nm.^54^^,^^55^ Additionally, heat dissipation into the tissue could be reduced by the addition of an active heat transport system (such as a microchannel heat sink) to the device architecture.^56^^,^^57^

The cost of the device will depend on the final choice of materials, the fabrication processes required, and the extent to which existing semiconductor fabrication pipelines are capable of meeting the requirements. Broadly, these devices can be fabricated with methods widely used in the nanofabrication field, but not all of these methods are industrialized at the scale of modern microchip manufacturing. Ultimately, such a device could be packaged together with optical or electrical stimulation channels for bidirectional neural interfacing.

If appropriate materials combinations can be fabricated, we have shown that the device could achieve the requisite sensitivity, noise level, and response time for recording both neural spikes and local field potentials. More broadly, our results suggest that integrated photonics could enable highly multiplexed readout of neuronal electrical signals via purely optical channels.
